# Supplementary material for: A fast-charging/discharging and long-term stable artificial electrode enabled by space charge storage mechanism
Source: Nat Commun. 2024 May 6;15:3778. doi: 10.1038/s41467-024-48215-2 (PMC11074309; doi:10.1038/s41467-024-48215-2)
Supplement: Supplementary file 1 — Supplementary Information [file 41467_2024_48215_MOESM1_ESM.docx]

**Supplementary information**

**A** **fast-charging/discharging and** **long-term stable artificial** **electrode enabled by** **space charge storage mechanism**

Linyi Zhao^1,4^, Tiansheng Wang^1,4^, Fengkai Zuo^1^, Zhengyu Ju^2^, Yuhao Li^1^, Qiang Li^1^, Yue Zhu^2,3^*, Hongsen Li^1^*, Guihua Yu^2^*

^1^College of Physics, Qingdao University, Qingdao 266071, China.

^2^Materials Science and Engineering Program and Walker Department of Mechanical Engineering, The University of Texas at Austin, Austin, Texas 78712, USA.

^3^School of Materials Science and Engineering, Ocean University of China, Qingdao 266404, China.

^4^These authors contributed equally to this work.

*Corresponding author Y.Z, H.L. and G.Y. (E-mails: zhuyue@ouc.edu.cn, hsli@qdu.edu.cn, ghyu@austin.utexas.edu).

Supplementary Notes

**Supplementary Note 1: The ionic and electronic conductivities of the Fe/Li_2_O.**

We measured the electronic and ionic conductivity using cold-pressing pellets and following the well-established AC and DC methods^1^. Due to its air sensitive nature, preparation of the sample and all subsequent measurements were conducted under an inert gas atmosphere. A VICTOR 4090A LCR digital bridge was used for these tests.

Theoretically, the measured impedance in AC mode (frequency of 100-1000 Hz) would be the combined impedance of electronic and ionic conduction. Whereas in DC mode, the contribution of ionic conductivity would rapidly decrease (we utilized a Cu electrode, which acts as a blocking electrode to the Li^+^ in DC mode). Thus, the stable results in DC mode would only reflect the contribution from electronic conductivity. As the measurement results in AC mode represent the mixed electronic and ionic conductivity, the ionic conductivity can be calculated by subtracting the electronic conductivity obtained in DC mode.

It should be noted that the preparation of the test specimen by cold-pressing may result in loosely-bound grains and possibly numerous voids, which could cause instability in the test results. To minimize such errors, we conducted multiple measurements and obtained the average values. The final test results are presented in Supplementary Table S2. The calculated average electronic conductivity of the Fe/Li_2_O material is 3.43×10^-6^ S m^-1^, while its ionic conductivity is 1.62×10^-5^ S m^-1^ (the electrical conductivity of the Fe/Li_2_O material is 1.96×10^-5^ S m^-1^). Again, due to the restriction of the cold-pressing method and the extremely small size of the constituting components in the material, these values could only serve as a rough estimation (lower limit) of the conductivities.

**Supplementary Note 2: Fabrication and performance evaluation of the Fe/Li_2_O||LiFePO_4_ full cell.**

The Fe/Li_2_O was paired with a LiFePO_4_ cathode to make a full cell. The cathode electrode slurry was prepared by mixing LiFePO_4_ (AR, Macklin, Shanghai, China), Super-P and PVDF binder dissolved in N-methyl-2-pyrrolidone (NMP) with the weight ratio of 80:10:10. The slurry was pasted onto an aluminum foil and the cathode was cut into 11-mm diameter discs with LiFePO_4_ active material loading of ~5.5 mg cm^-2^. A Celgard 2325 film (Whatman) and the 1 M LiPF_6_ in 1:1 (volume ratio) ethylene carbonate (EC) and diethyl carbonate (DEC) was used as separator and electrolyte, respectively. The volume of the electrolyte was ~90 μL. The Fe/Li_2_O were firstly electrochemical prelithiation in a half-cell configuration (vs metal Li) before used as anode in full cells. The CR2032-type full cells were assembled in a glove box filled with argon gas with the LiFePO_4_ electrode as cathode and the prelithiation Fe/Li_2_O electrode as anode. Galvanostatic charge/discharge measurements were performed between 1.0 to 3.2 V at the current density of 100 mA g^-1^. Specific capacities were calculated based on the cathode materials of each electrode.

The negative-to-positive (N/P) electrode capacity ratio was around 1.1 for the Fe/Li_2_O||LiFePO_4_ full cell. The initial charge voltage curve of the Li||LiFePO_4_ half-cell under 100 mA g^-1^ and the discharge voltage curve of the Li||LiFePO_4_ half-cell under 100 mA g^-1^ were used to simulate the charge voltage profile of a Fe/Li_2_O||LiFePO_4_ full cell. LiFePO_4_ has an initial charge specific capacity of 0.94 mAh while Fe/Li_2_O delivers 1.03 mAh upon discharge to 0.1 V versus Li/Li^+^. The voltage range of Fe/Li_2_O is 0.1-3.0 V and its capacity is normalized to 1.1 times the LiFePO_4_ capacity. The mass loading of the cathode and anode were controlled to realize a negative-to-positive electrode capacity ratio of 1.1 (Supplementary Fig. S2a). The rate capability of the Fe/Li_2_O||LiFePO_4_ full cell is shown in Supplementary Fig. S2b, the reversible specific capacities of 166, 127, 110, 100 and 93 mAh g^-1^ at current densities of 0.1, 0.2, 0.3, 0.4 and 0.5 A g^-1^ are obtained, respectively. The capacity of the Fe/Li_2_O||LiFePO_4_ full cell after the 500th cycle is 140 mAh g^-1^, resulting in a capacity retention of 85 % and an average coulombic efficiency of >96 % (Supplementary Fig. S2c and S2d).

**Supplementary Note 3: Calculation method for power density.**

To compare Fe/Li_2_O electrodes with previously reported high-rate anode materials, a Ragone plot is shown in Supplementary Fig. S3. Its specific energy density (*E*, Wh kg^-1^) and power density (*P,* kW kg^-1^) are calculated from the equations as follows:

$$\begin{aligned} P=\frac{Q\Delta E}{m\Delta t}=\frac{i\Delta E}{m}\#\left( 1 \right) \end{aligned}$$

$$\begin{aligned} E=P\Delta t\#\left( 2 \right) \end{aligned}$$

Where $\Delta E=\frac{E_{max}+E_{min}}{2}$. In these equations, Q(mAh), *i*(A), m(g), and Δ*t*(s) denote the charge delivered during discharge, discharge current, mass of active materials, and discharge time, respectively. *E*_max_ and *E*_min_ are the initial and final potentials of discharge curves of galvanostatic cycling at different current densities^2–4^. It can be seen that Fe/Li_2_O exhibits higher energy densities for the full power density range outperforming all other reported materials (Supplementary Fig. S3a). Even at a power density as high as 113.4 kW kg^-1^, it still retains an energy density of 189 Wh kg^-1^.

Supplementary Fig. S3b represents the Ragone plot of the Fe/Li_2_O||LiFePO_4_ full cell and other previously reported high-rate hybrid devices. The specific energy density (*E*, Wh kg^-1^) and power density (*P,* kW kg^-1^) are calculated by the aforementioned equations (1) and (2). It can be seen that the full cell achieves a maximal high-level energy density of 194.4 Wh kg^-1^ at 8.4 kW kg^-1^. The full cell shows energy preponderance when compared with many excellent reported devices.

Supplementary Tables

**Table S1 |** ICP results of all the synthesized samples in this study.

| Tested material | Mass fraction of Fe element  (Experimental value) | Mass fraction of Fe element  (Theoretical value) |
| --- | --- | --- |
| Fe/Li_2_O | 40 % | 53 % |
| Fe/LiF | 32 % | 38 % |
| Fe/Li_2_S | 41 % | 48 % |
| Fe/Li_3_N | 61 % | 69 % |

**Table S2.** The test results for conductivity in AC mode and DC mode of the material.

|  | Resistance  (R, Ω) | Resistivity  (ρ, Ω·m) | Conductivity  (σ, S·m^-1^) | Tablet diameter  (ф, mm) | Tablet thickness  (L, mm) |
| --- | --- | --- | --- | --- | --- |
| AC mode | 5.09×10^6^ | 5.95×10^4^ | 1.68×10^-5^ | 5.00 | 1.68 |
|  | 4.02×10^6^ | 4.70×10^4^ | 2.13×10^-5^ | 5.00 | 1.68 |
|  | 4.32×10^6^ | 5.05×10^4^ | 1.98×10^-5^ | 5.00 | 1.68 |
|  | 3.76×10^6^ | 4.40×10^4^ | 2.27×10^-5^ | 5.00 | 1.68 |
|  | 4.83×10^6^ | 5.64×10^4^ | 1.77×10^-5^ | 5.00 | 1.68 |
| DC mode | 2.49×10^7^ | 2.93×10^5^ | 3.42×10^-6^ | 5.00 | 1.68 |
|  | 2.40×10^7^ | 2.80×10^5^ | 3.57×10^-6^ | 5.00 | 1.68 |
|  | 2.42×10^7^ | 2.83×10^5^ | 3.54×10^-6^ | 5.00 | 1.68 |
|  | 2.51×10^7^ | 2.94×10^5^ | 3.40×10^-6^ | 5.00 | 1.68 |
|  | 2.65×10^7^ | 3.10×10^5^ | 3.23×10^-6^ | 5.00 | 1.68 |

**Table S3 |** Detailed information of the relevant reports of fast charging materials in terms of capacity retention (CR), specific capacity at high rate (SCH), specific capacity at low rate (SCL), rate ability (Rat.), cycle ability (Cyc.) and final capacity (FC).

| Materials | CR  (%) | SCH  (mAh g^-1^) | SCL  (mAh g^-1^) | Rat.  (C) | Cyc.  (number) | FC  (mAh g^-1^) | Reference |
| --- | --- | --- | --- | --- | --- | --- | --- |
| lithium yttrium titanate (LYTO) | 98 | 87 | 210 | 100 | 3000 | 180 | [5] |
| Rock salt L_3_V_2_O_5_ | 87 | 109 | 266 | 200 | 1000 | 133 | [6] |
| TiO_2_/3D Cu-GM | 88 | 94 | 284 | 60 | 1000 | 83 | [7] |
| NiNb_2_O_6_ | 78 | 50 | 244 | 100 | 20000 | 47 | [8] |
| Li_4_Ti_5_O_12_  (LTO-600) | 88 | 146 | 181 | 100 | 1500 | 150 | [9] |
| TiF_3_(Ti/LiF) | 59 | 44 | 64 | 6 | 15 | 240 | [10,11] |

**Table S4 |** The loading of active material of some anode materials for lithium-ion batteries in the research field of fast charging.

| Anode material | Mass loading | References |
| --- | --- | --- |
| lithium yttrium titanate (LYTO) | 1.5 mg cm^-2^ | [5] |
| Rock salt L_3_V_2_O_5_ | 2~3 mg cm^-2^ | [6] |
| TiO_2_/3D Cu-GM | 0.537 mg cm^-2^ | [7] |
| NiNb_2_O_6_ | 1~3 mg cm^-2^ | [8] |
| Li_4_Ti_5_O_12_  (LTO-600) | 2.0 mg cm^-2^ | [9] |

**Supplementary Figures**


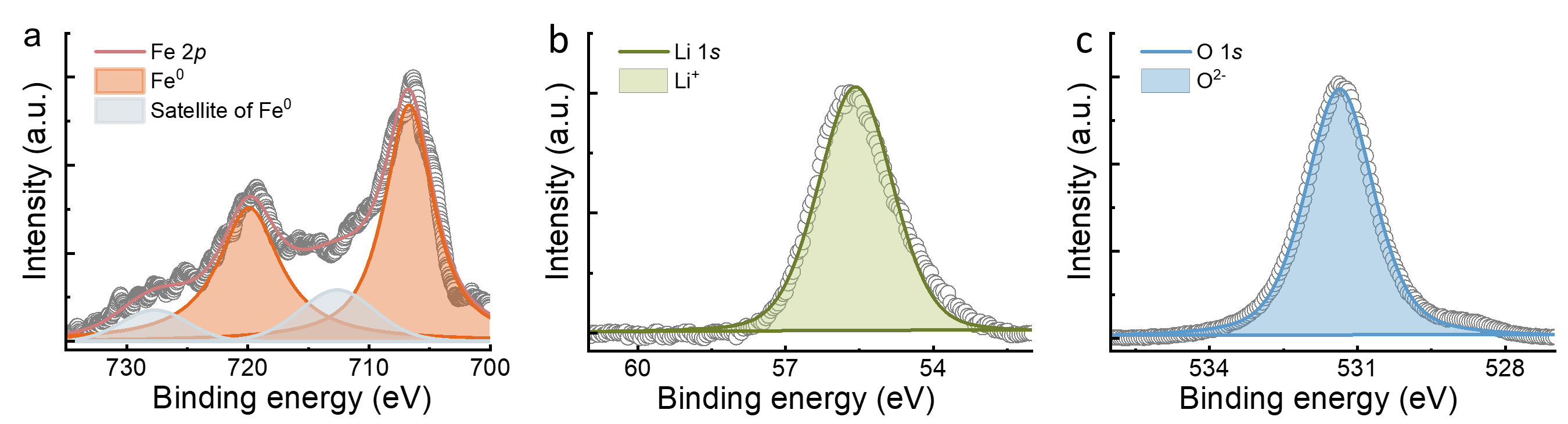


**Supplementary Fig. S1 |** High-resolution XPS spectra of different elements of the as-synthesized Fe/Li_2_O, corresponding to Fe 2*p* (**a**) Li 1*s* (**b**) and O 1*s* (**c**), respectively.


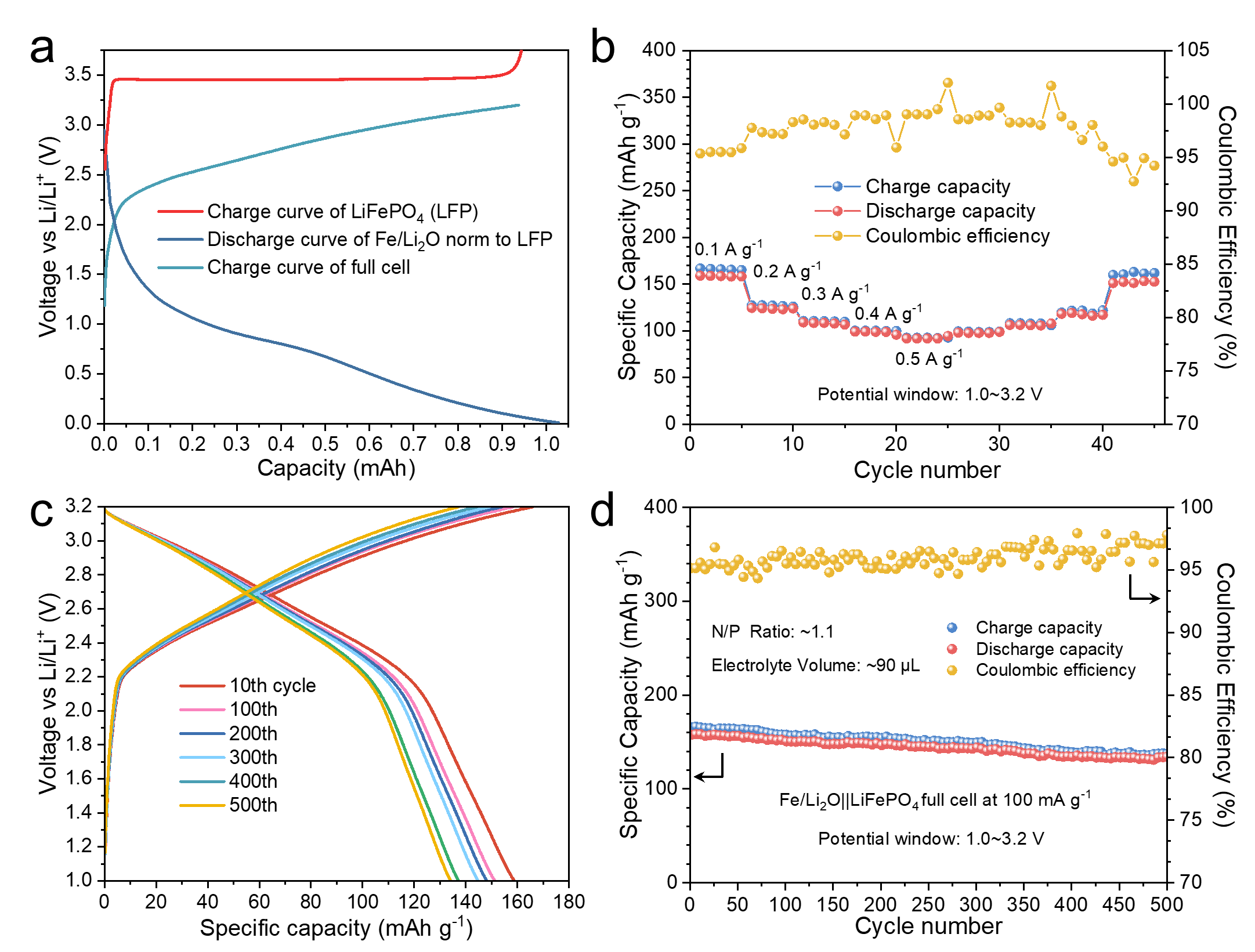


**Supplementary Fig. S2 |** The electrochemical performance of Fe/Li_2_O||LiFePO_4_ full cell. (**a**) The initial charge voltage curve of a Li||LiFePO_4_ half-cell under 100 mA g^-1^ and the discharge voltage curve of a Li||Fe/Li_2_O half-cell under 100 mA g^-1^ were used to assemble Fe/Li_2_O||LiFePO_4_ full cell. The negative-to-positive electrode capacity ratio was around 1.1. (**b**) Rate capability of Fe/Li_2_O||LiFePO_4_ full cell at different rates (from 0.1 to 0.5 A g^-1^). (**c**) Voltage profiles over the course of 500 cycles for a voltage window of 1.0–3.2 V. (**d**) The cycling stability of the Fe/Li_2_O||LiFePO_4_ full cell at 100 mA g^-1^ for 500 cycles.


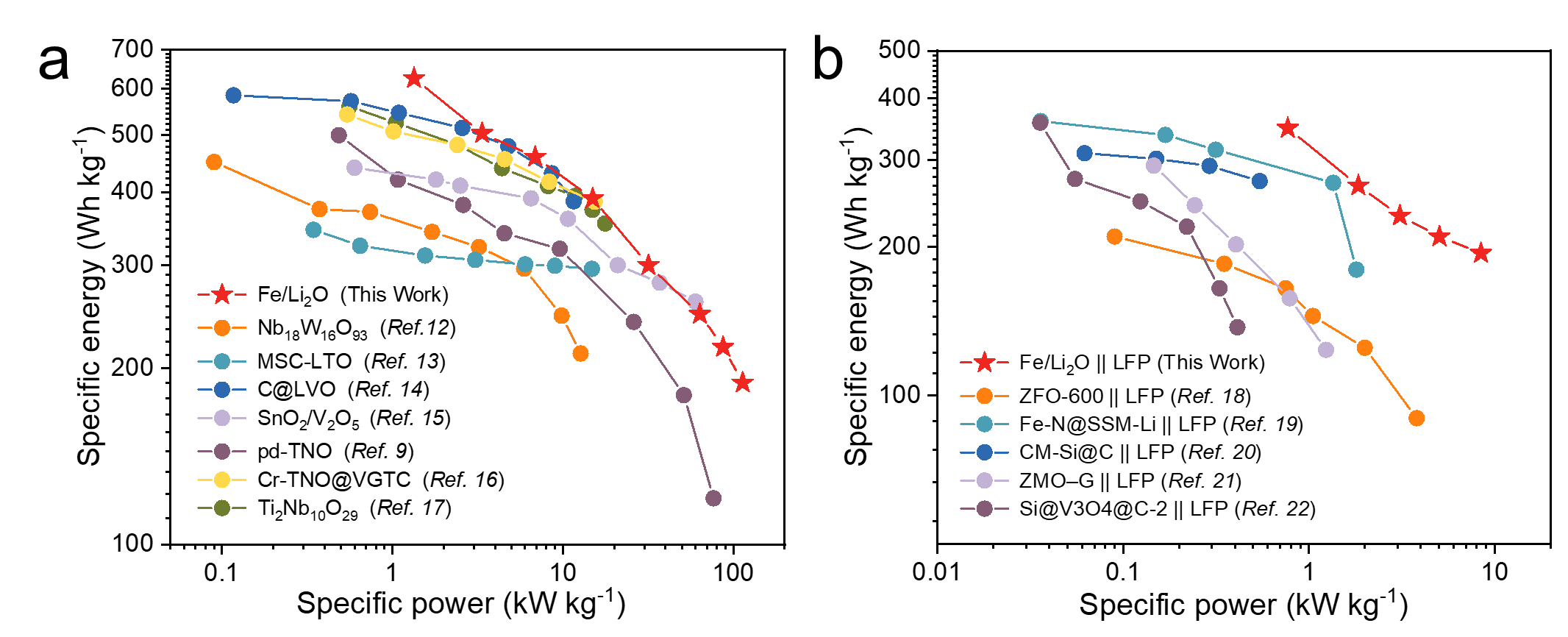


**Supplementary Fig. S3 |** Ragone plot comparing Fe/Li_2_O electrode (**a**) and Fe/Li_2_O||LiFePO_4_ full cell (**b**) to published results in the similar fields^9,12–22^.


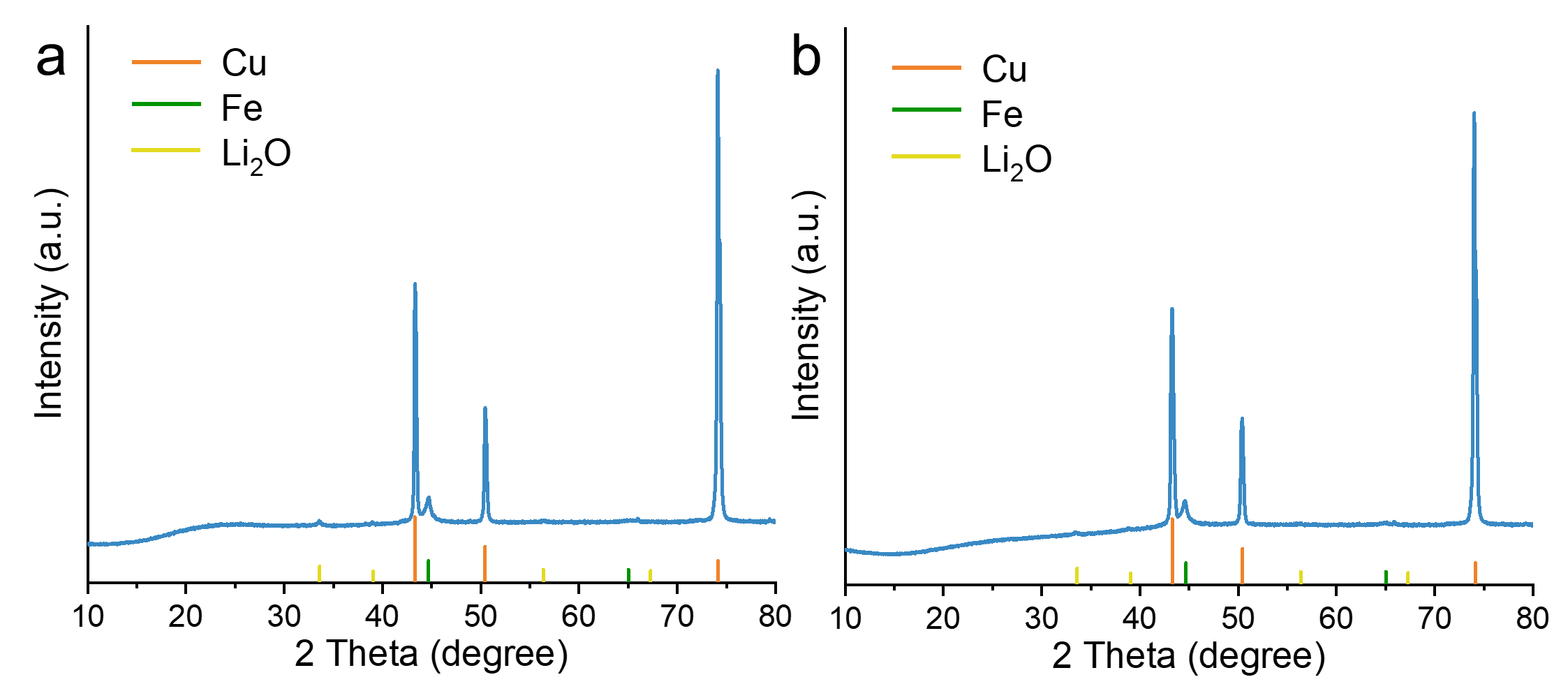


**Supplementary Fig. S4 |** XRD patterns of the Fe/Li_2_O electrode when discharged to 0.01 V (**a**) and charged to 3 V (**b**) in lithium-ion batteries.


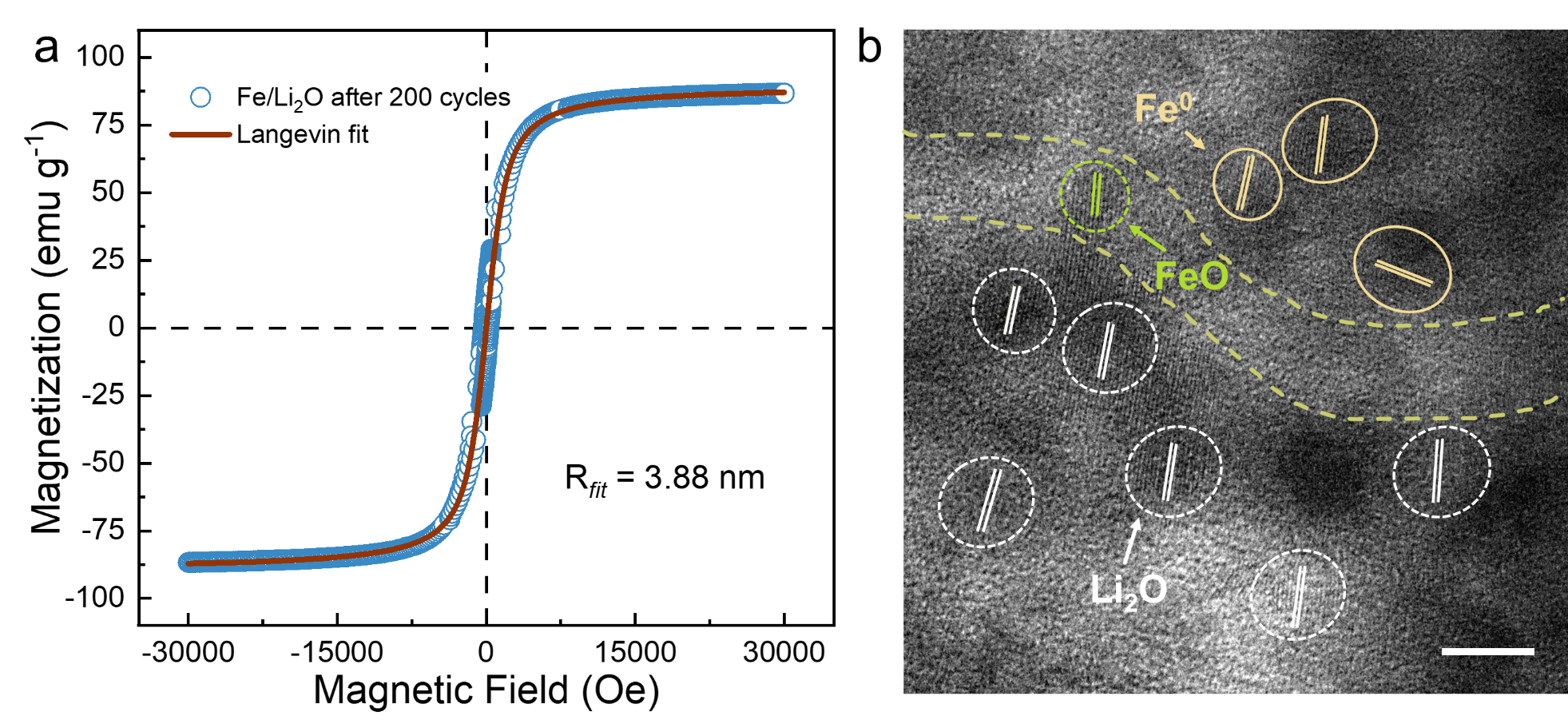


**Supplementary Fig. S5 |** The M-H curve and HRTEM image of the Fe/Li_2_O after 200 cycles. (**a**) M-H curve of the Fe/Li_2_O at room temperature and the corresponding Langevin fitting curve after 200 cycles at a current density of 1 A g^-1^. (**b**) HRTEM image of the Fe/Li_2_O when charged to 3 V after cycling for 200 cycles at a current density of 1 A g^-1^. Scale bar is 5 nm.


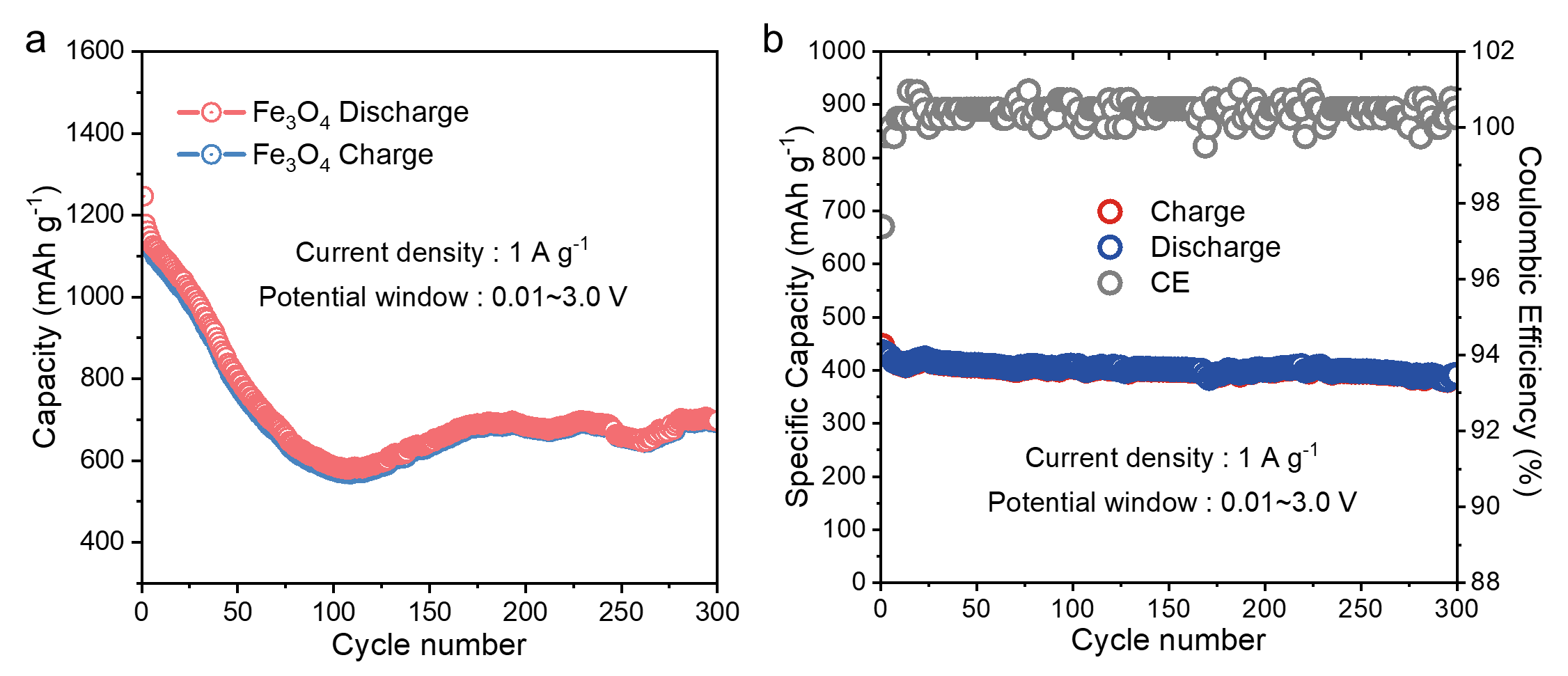


**Supplementary Fig. S6 |** Comparison of the cycling performance between Fe_3_O_4_ and Fe/Li_2_O. **(a)** Cycling curves of the Fe_3_O_4_ at the current density of 1 A g^-1^ for the first 300 cycles. **(b)** Cycling curves of the Fe/Li_2_O electrode at the current density of 1 A g^-1^ for the first 300 cycles.


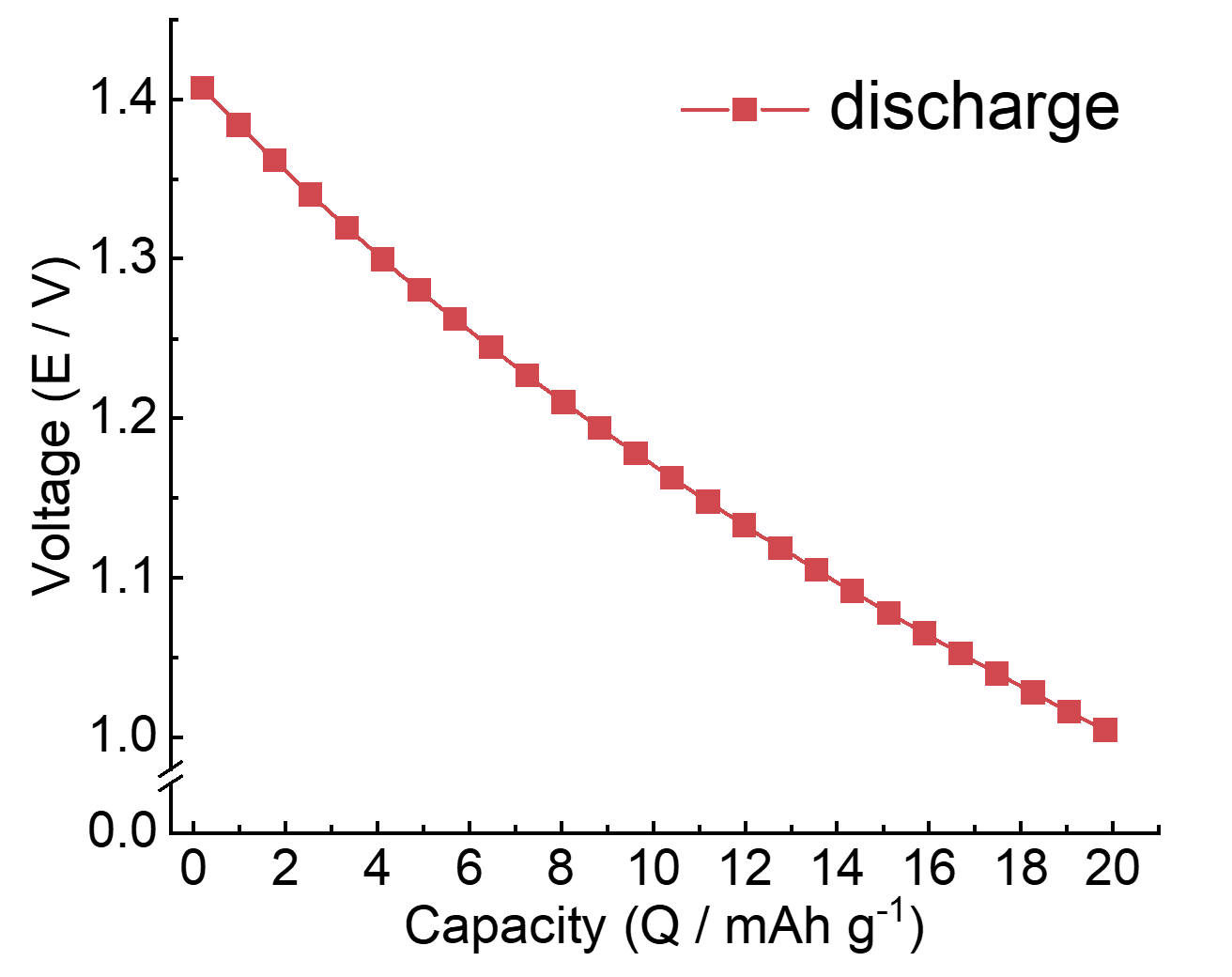


**Supplementary Fig. S7 |** The open-circuit voltage of a battery with Fe/Li_2_O as the cathode and Li metal as the anode as a function of specific capacity.


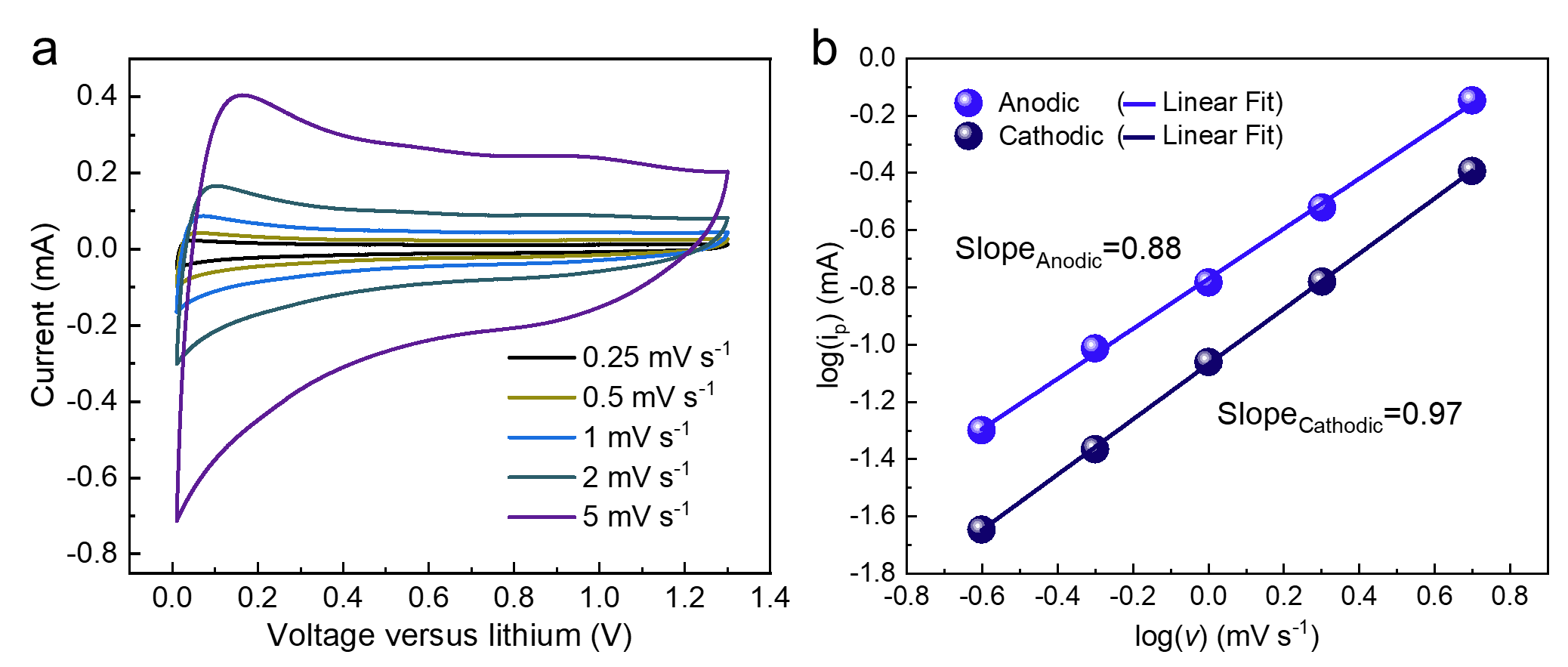


**Supplementary Fig. S8 |** Electrochemical property of the Fe/Li_2_O electrode in the potential window of 0.01-1.3 V. (**a**) CV curves of the Fe/Li_2_O electrode at different scan rates in the voltage range of 0.01-1.3 V. (**b**) b values calculated by the relationship between peak current and scan rate in CV curves.


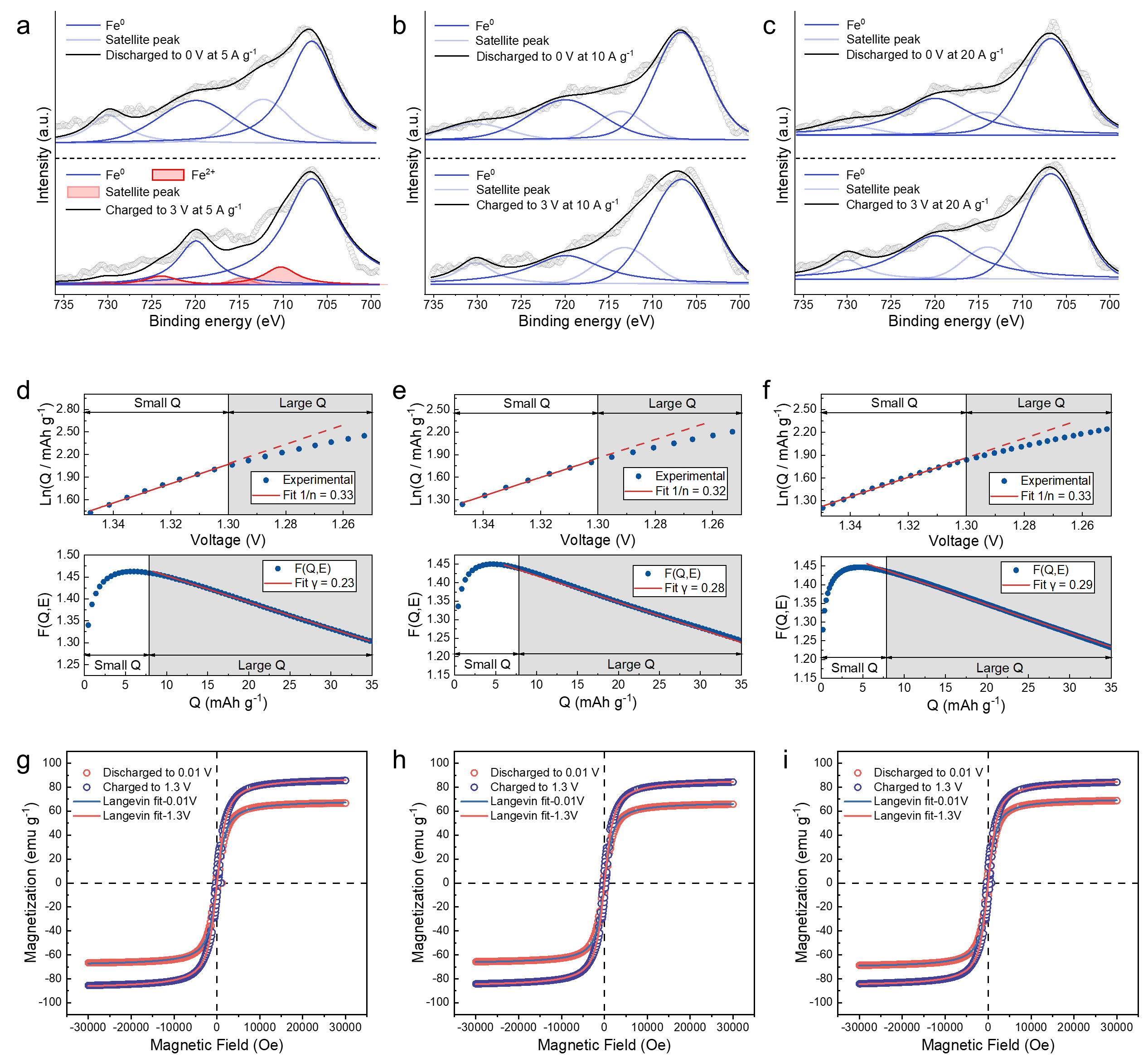


**Supplementary Fig. S9** **|** High-resolution XPS spectra of the Fe element in the Fe/Li_2_O electrode at different charge and discharge potentials with a current density of 5 A g^-1^ (**a**), 10 A g^-1^ (**b**), and 20 A g^-1^ (**c**), respectively. Ln(Q)-V fitting curve and F(Q,E)-Q fitting curve of the Fe/Li_2_O electrode at the current density of 5 A g^-1^ (**d**), 10 A g^-1^ (**e**), and 20 A g^-1^ (**f**), respectively. M-H curves of the electrode discharged to 0.01 V and charged to 1.3 V at room temperature and the corresponding Langevin fitting curves at the current density of 5 A g^-1^ (**g**), 10 A g^-1^ **h**), and 20 A g^-1^ (**i**), respectively.

**
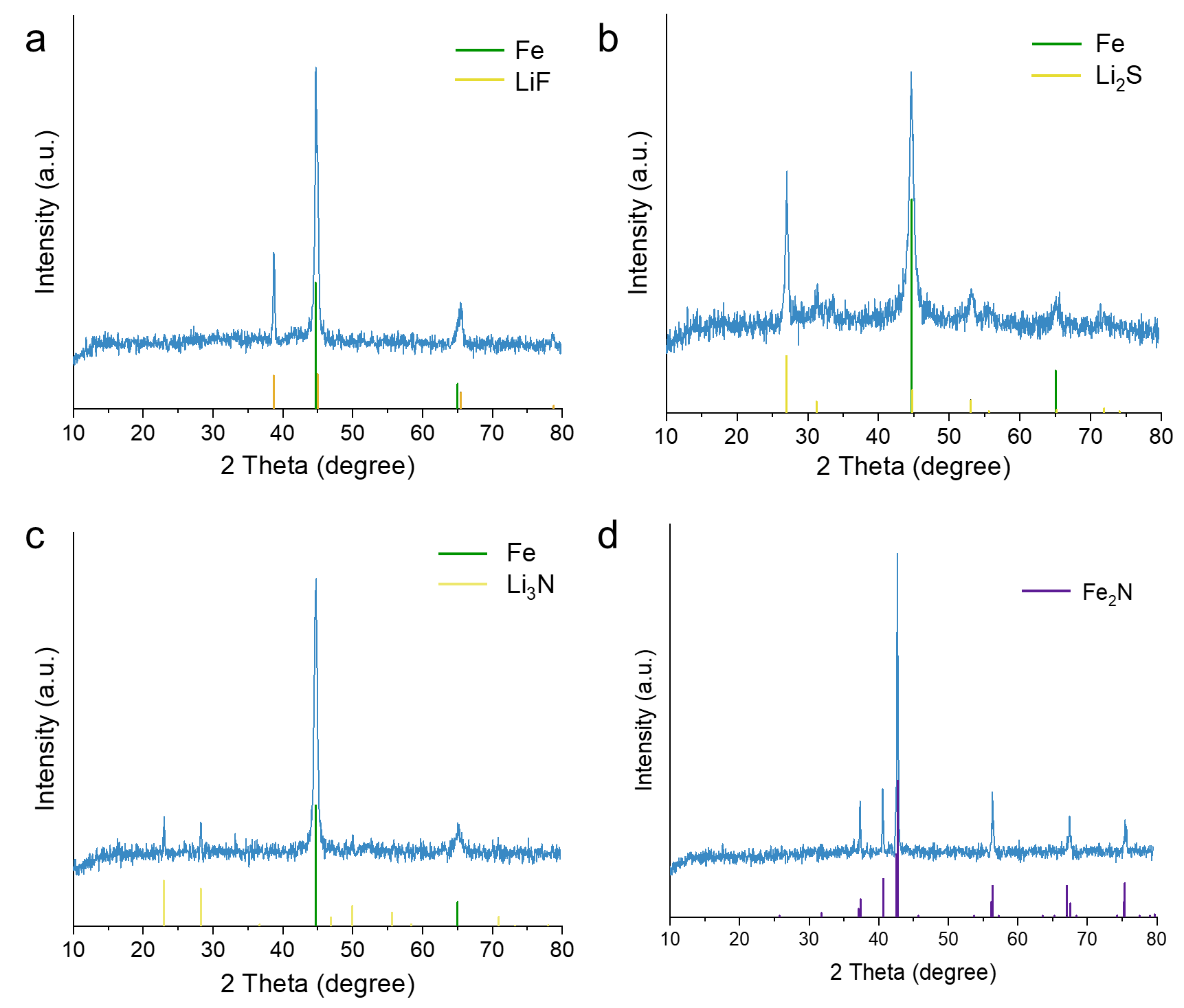
**

**Supplementary Fig. S10** **|** XRD diffraction patterns of the chemically synthesized Fe/LiF (**a**) Fe/Li_2_S (**b**) Fe/Li_3_N (**c**) and the Fe_2_N precursor used in producing Fe/Li_3_N (**d**). The corresponding PDF standard cards are LiF: JCPDS No.12-0254; Li_2_S: JCPDS No.23-0369; Li_3_N: JCPDS No.30-0759 and Fe_2_N: JCPDS No.50-0958.


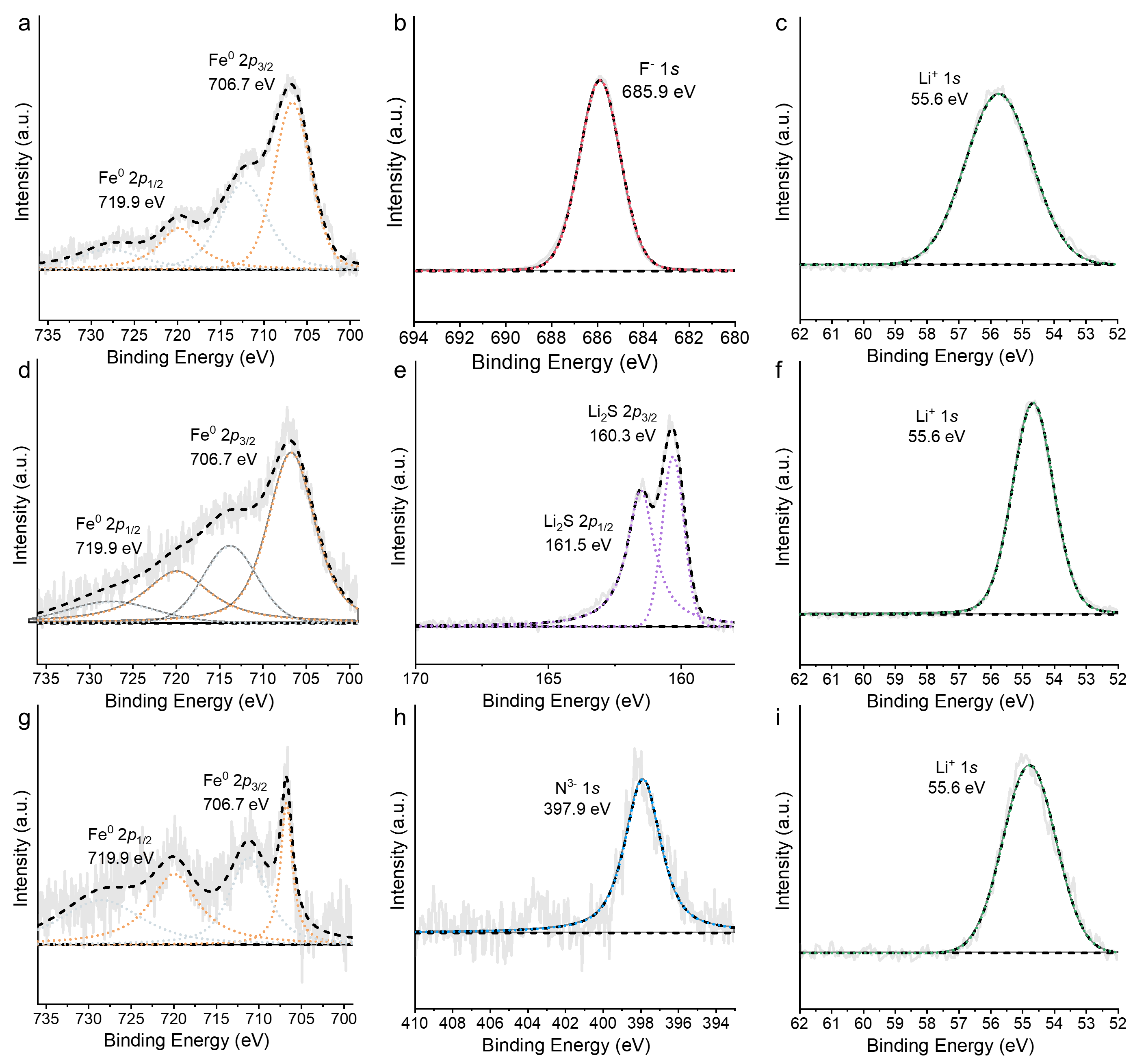


**Supplementary Fig. S11** **|** XPS spectra of the chemically synthesized Fe/Li_x_M (M=F, S, N). **a-c**, High-resolution XPS spectra of elements in Fe/LiF, corresponding to Fe 2*p* (**a**), F 1*s* (**b**), and Li 1*s* (**c**), respectively. **d-f**, High-resolution XPS spectra of elements in Fe/Li_2_S, corresponding to Fe 2*p* (**d**), S 2*p* (**e**), and Li 1*s* (**f**), respectively. **g-i**, High-resolution XPS spectra of elements in Fe/Li_3_N, corresponding to Fe 2*p* (**g**), N 1*s* (**h**), Li 1*s* (**i**), respectively.

**
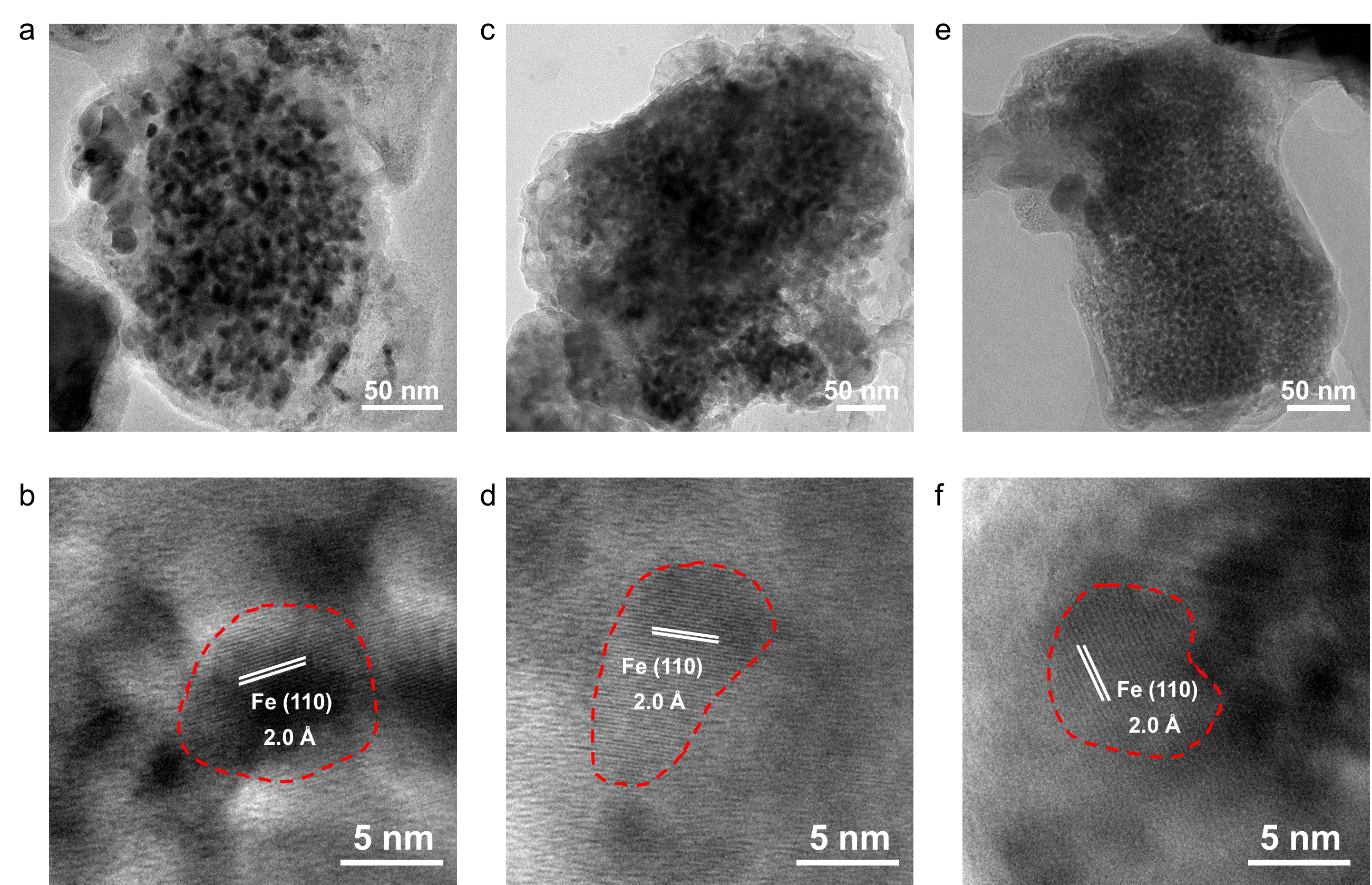
**

**Supplementary Fig. S12** **|** HRTEM image of the chemically synthesized Fe/Li_x_M (M=F, S, N). **a-b,** TEM image (**a**) and HRTEM image (**b**) of Fe/LiF. **c-d,** TEM image (**c**) and HRTEM image (**d**) of Fe/Li_2_S. **e-f**, TEM image (**e**) and HRTEM image (**f**) of Fe/Li_3_N.


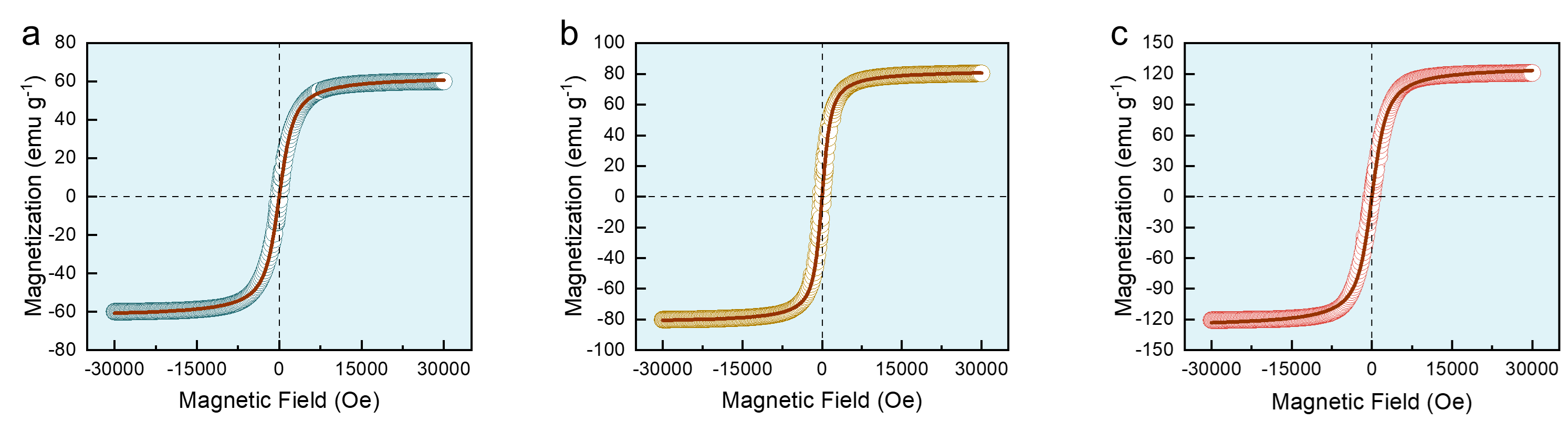


**Supplementary Fig. S13** **|** M-H curves and Langevin fitting curves of the Fe/LiF (**a**), Fe/Li_2_S (**b**) and Fe/Li_3_N (**c**).

**References**

1. Huggins, R. A. Simple method to determine electronic and ionic components of the conductivity in mixed conductors a review. *Ionics* **8**, 300–313 (2002).

2. Que, L., Wang, Z., Yu, F. & Gu, D. 3D ultralong nanowire arrays with a tailored hydrogen titanate phase as binder-free anodes for Li-ion capacitors. *J. Mater. Chem. A* **4**, 8716–8723 (2016).

3. Aravindan, V., Shubha, N., Ling, W. C. & Madhavi, S. Constructing high energy density non-aqueous Li-ion capacitors using monoclinic TiO_2_-B nanorods as insertion host. *J. Mater. Chem. A* **1**, 6145 (2013).

4. Han, X. *et al.* Nitrogen-doped carbonized polyimide microsphere as a novel anode material for high performance lithium ion capacitors. *Electrochim. Acta* **196**, 603–610 (2016).

5. Zhang, Y. *et al.* Layered perovskite lithium yttrium titanate as a low‐potential and ultrahigh‐rate anode for lithium‐ion batteries. *Adv. Energy Mater.* **12**, 2200922 (2022).

6. Liu, H. *et al.* A disordered rock salt anode for fast-charging lithium-ion batteries. *Nature* **585**, 63–67 (2020).

7. Hyun, G. *et al.* Three-dimensional, submicron porous electrode with a density gradient to enhance charge carrier transport. *ACS Nano* **16**, 9762–9771 (2022).

8. Xia, R. *et al.* Nickel niobate anodes for high rate lithium‐ion batteries. *Adv. Energy Mater.* **12**, 2102972 (2021).

9. Wu, W. *et al.* Unprecedented superhigh‐rate and ultrastable anode for high‐power battery via cationic disordering. *Adv. Energy Mater.* **12**, 2201130 (2022).

10. Li, H., Balaya, P. & Maier, J. Li-storage via heterogeneous reaction in selected binary metal fluorides and oxides. *J. Electrochem. Soc.* **151**, A1878 (2004).

11. Yu, X. Q. *et al.* Reversible lithium storage in LiF/Ti nanocomposites. *Phys. Chem. Chem. Phys.* **11**, 9497 (2009).

12. Griffith, K. J., Wiaderek, K. M., Cibin, G., Marbella, L. E. & Grey, C. P. Niobium tungsten oxides for high-rate lithium-ion energy storage. *Nature* **559**, 556–563 (2018).

13. Jin, X. *et al.* Mesoporous Single‐crystal lithium titanate enabling fast‐charging Li‐ion batteries. *Adv. Mater.* **34**, 2109356 (2022).

14. Shen, L., Chen, S., Maier, J. & Yu, Y. Carbon‐coated Li_3_VO_4_ spheres as constituents of an advanced anode material for high‐rate long‐life lithium‐ion batteries. *Adv. Mater.* **29**, 1701571 (2017).

15. Yan, J., Sumboja, A., Khoo, E. & Lee, P. S. V_2_O_5_ loaded on SnO_2_ nanowires for high‐rate Li ion batteries. *Adv. Mater.* **23**, 746–750 (2011).

16. Deng, S. *et al.* Synergy of ion doping and spiral array architecture on Ti_2_Nb_10_O_29_ : a new way to achieve high‐power electrodes. *Adv. Funct. Mater.* **30**, 2002665 (2020).

17. Yao, Z. *et al.* Superior high-rate lithium-ion storage on Ti_2_Nb_10_O_29_ arrays via synergistic TiC/C skeleton and N-doped carbon shell. *Nano Energy* **54**, 304–312 (2018).

18. Varzi, A., Bresser, D., Von Zamory, J., Müller, F. & Passerini, S. ZnFe_2_O_4_‐C/LiFePO_4_‐CNT: A novel high‐power lithium‐ion battery with excellent cycling performance. *Adv. Energy Mater.* **4**, 1400054 (2014).

19. Fu, X., Duan, H., Zhang, L., Hu, Y. & Deng, Y. A 3D framework with an in situ generated Li_3_N solid electrolyte interphase for superior lithium metal batteries. *Adv. Funct. Mater.* **33**, 2308022 (2023).

20. Wang, B., Li, W., Wu, T., Guo, J. & Wen, Z. Self-template construction of mesoporous silicon submicrocube anode for advanced lithium ion batteries. *Energy Storage Mater.* **15**, 139–147 (2018).

21. Xiong, P. *et al.* Two-dimensional nanosheets based Li-ion full batteries with high rate capability and flexibility. *Nano Energy* **12**, 816–823 (2015).

22. Wang, Z. *et al.* Rational design of ion‐conductive layer on Si anode enables superior‐stable lithium‐ion batteries. *Small* **20**, 2306428 (2024).
